# Comparison of Research Framing Preferences and Information Use of State Legislators and Advocates Involved in Cancer Control, United States, 2012–2013

**DOI:** 10.5888/pcd14.160292

**Published:** 2017-02-02

**Authors:** Alexandra B. Morshed, Elizabeth A. Dodson, Rachel G. Tabak, Ross C. Brownson

**Affiliations:** 1 Prevention Research Center in St. Louis, Brown School, Washington University in St. Louis, St. Louis, Missouri; 2 Department of Surgery and Alvin J. Siteman Cancer Center, Washington University School of Medicine, Washington University in St. Louis, St. Louis, Missouri

## Abstract

**Introduction:**

Evidence-based policy plays an important role in prevention of cancer and other chronic diseases. The needs of actors involved in policy decision-making should inform knowledge translation strategies. This study examines the differences between state legislators and advocates in how they seek and use information and what their preferences are for how research information is framed.

**Methods:**

We conducted a cross-sectional comparison of survey responses by US advocates (n = 77) and state legislators (n = 265) working on issues related to cancer control.

**Results:**

Advocates differed significantly from legislators on all demographic characteristics. Advocates reported seeking and using information more frequently than legislators, though legislators used legislative research bureaus more often (0.45 point difference, *P* = .004). Both legislators and advocates prioritized the presentation and timeliness of research information similarly but reported different preferences for source (information bias, information relevance, delivery of information by trusted person) of research information. Several differences between advocates and legislators were modified by participant age.

**Conclusion:**

Our study provides insights for development of knowledge translation strategies to enhance evidence-based policy making for cancer control that are tailored to state-level legislators and advocates. Additional research efforts should evaluate the effectiveness of such knowledge translation strategies, particularly among advocates.

## Introduction

Enactment of evidence-based legislation can produce considerable population effects on preventable conditions such as cancer and its associated risk factors (1,2). Nonetheless, public health policy making often is not informed by available evidence (3,4). Kingdon’s Multiple Streams Framework of how policy change happens defines the policy stream as the space where solutions compete for acceptance during decision making around an agreed-upon problem (5), such as cancer control. As researchers seek to translate cancer control knowledge into policy solutions within this stream, they must consider the needs of actors involved in policy decision making around control of cancer and its risk factors and frame their messages accordingly (6,7).

Policy makers value and seek scientific research information (8,9), and they cite needs of constituents; collaboration and relationships; clarity, relevance, and reliability of research findings; and timeliness and opportunity as key factors in using research in policy making (9). Though people from the same institutions often share some preferences for research framing, differences exist among people involved in policy making (eg, legislators, legislative staff, agency officials) and should be explored (8,10).

Policy advocates are less often the focus of dissemination research though they play an important role in the policy making process. Advocacy entails the use of information, resources, and skills to create changes in the public’s opinion and views of decision makers, which then influence public policies or the policy process (11,12). Advocates are often channels of communication to policy makers and routinely build long-term relationships with them (13). Given their role in evidence-based policy making (14), it is important to examine how researchers should tailor messages to policy advocates.

This study considers differences in how legislators and advocates involved in policy work concerning cancer and its risk factors seek and use information during policy decision making and what their preferences are for how research information is framed.

## Methods

### Design and participants

We conducted a cross-sectional comparison of US advocates and state legislators who work on issues related to cancer or cancer-related risk factors. Seventy-seven advocates and 265 legislators participated in the study. We identified the initial sample of advocates by conducting a Google search of the following key words: *advocacy, policy, obesity, physical activity, cancer,* or *nutrition* and combined these key words with the state name (eg, California). We used the policyexperts.org and heritage.org Web-based advocacy resources to identify more politically conservative advocacy groups to achieve adequate variability in the sample by political stance. In each advocacy agency, we defined the contact person as the individual who was responsible for public policy efforts or worked with government agencies. We verified the accuracy of contact information in the initial contact list (290 contacts) and made corrections where possible, resulting in a final list of 213 contacts. From February through April 2013, people on the contact list were repeatedly contacted; telephone numbers for people we were unable to reach were returned to the queue and routinely redialed until the end of the data collection period, and trained personnel conducted telephone interviews with advocates who agreed to participate. Additional information regarding recruitment procedures is available elsewhere (15).

To obtain the legislator sample, we partnered with the National Conference of State Legislatures to compile a complete list of all 7,525 state legislators from 50 states and 3 US territories. A random sample of 2,000 legislators was selected from this population, and each legislator was contacted up to 10 times for participation in the study. Those who agreed to participate completed interviews by telephone from January through October 2012. Of those legislators contacted, 862 completed the survey, and 161 started but did not complete it; 857 legislators declined to participate.

To ensure comparability with the advocate sample, we retained for analysis the legislators who indicated that cancer or cancer risk factors (tobacco use, diet/nutrition, physical activity, obesity) were among their legislative priorities. We also focused on this group of legislators, because they were more likely to already work on policies related to cancer control or serve on related legislative committees. We based this selection on responses to 2 survey questions: 1) an open-ended question asking, “What issues are your legislative priorities?” and 2) a question asking participants to choose their most important health issues from a 19-item list where the last item (“other”) was also open-ended. If the legislator selected cancer, diet/nutrition, physical activity, obesity, or tobacco use/cessation in the second question, they were included in the study. The open-ended responses were also screened for the terms *cancer, diet, nutr, activ, exercise, obes, smok, *and* tobacco*, and 2 independent coders reviewed these participant responses, resolved contrasting coding decisions through discussion, and coded the responses as prioritizing cancer or cancer risk factors or not prioritizing cancer or cancer risk factors. The 265 legislators selected through this process were included in the study. The institutional review board of Washington University in St. Louis approved this study.

### Measures

The main outcomes in our study were information seeking and use and preferences for how research information is framed. We adapted questions from a previously developed measure validated with state legislators and cognitively tested with representatives of the advocate sample (8,15–17). To measure information seeking and use, we asked participants to rank a series of statements on a 5-point scale (1 = never, 5 = always) on the basis of how often they used each source of information when working on policy issues. To measure preferences for how research information is framed, we asked participants to rank a series of statements on a 5-point scale (1 = low, 5 = high) indicating how important it was to the participant that research information have a given characteristic. The research information characteristics represented 3 research information domains: source, presentation, and timeliness (18).

In addition, we collected characteristics of participants: sex, age (<40 y, 40–49 y, 50–59 y, or ≥60 y), educational attainment (some college, trade, technical, or vocational education or less; college degree; or postgraduate degree), self-rated health status (excellent, very good, good, or fair or poor), and the number of years the participant had spent in the legislature or in advocacy. We also measured political stance by asking participants to rate themselves on social and fiscal issues and collapsing their answers into 3 categories: liberal (slightly liberal, liberal, extremely liberal), moderate, and conservative (slightly conservative, conservative, extremely conservative).

### Data analysis

We used Pearson’s χ^2^ to examine differences in group characteristics for categorical variables and Wilcoxon rank sum test for continuous variables. To examine the differences between legislators and advocates in information-seeking, information use, and preferences for framing of research information, we used a one-way analysis of variance. In addition, we examined whether age modified the differences between advocates and legislators by using the interaction term between type of actor (legislator, advocate) and age (<50 y, ≥50 y), and where significant, the analyses were stratified by age. We used SAS version 9.4 (SAS Institute, Inc) to conduct analyses.

## Results

The response rates for the legislator survey was 54%, and the response rate for the advocate survey was 36%. Advocates differed significantly from legislators on all characteristics (Table 1). More advocates were female (61.0%), whereas most legislators were male (61.7%). Advocates were younger than legislators and had higher educational attainment. Legislators rated their health status lower than advocates did and had more conservative views on social and fiscal issues.

**Table 1:** Characteristics of Participating Advocates (N = 77) and Legislators (N = 265) Involved in Cancer Control, Study of Information Use, Information-Seeking, and Research Framing Preferences, United States, 2012–2013^a^

Characteristic	Advocates	Legislators	*P* Value^b^
**Female**	47 (61.0)	75 (28.3)	<.001
**Age, y**			
<40	21 (27.6)	11 (5.5)	<.001
40–49	20 (26.3)	29 (14.4)	
50–59	15 (19.7)	51 (25.4)	
≥60	20 (26.3)	110 (54.7)	
**Education**			
Some college, trade, technical, or vocational education or less	1 (1.3)	46 (17.4)	<.001
College degree	28 (36.8)	105 (39.6)	
Postgraduate degree	47 (61.8)	114 (43.0)	
**Self-rated health**			
Excellent	33 (43.4)	61 (23.0)	.001
Very good	29 (38.2)	97 (36.6)	
Good	12 (15.8)	85 (32.1)	
Fair or poor	2 (2.6)	22 (8.3)	
**Political stance, social issues**			
Liberal	43 (65.2)	77 (29.3)	<.001
Moderate	10 (15.2)	49 (18.6)	
Conservative	13 (19.7)	137 (52.1)	
**Political stance, fiscal issues**			
Liberal	35 (49.3)	27 (10.2)	<.001
Moderate	15 (21.1)	58 (22.0)	
Conservative	21 (29.6)	179 (67.8)	
**Number of years in legislature or advocacy, mean (standard deviation)**	14.41 (9.88)	9.11 (7.67)	<.001

a Values expressed as no. (%) unless otherwise indicated.

b Differences were examined using Pearson’s χ^2^ for categorical variables and Wilcoxon rank sum test for the continuous variable.

We examined differences between advocates and legislators in their information seeking, information use, and preferences for framing of research information (Table 2). Advocates reported seeking and using information more frequently than did legislators, except for the “Ask research bureaus (external legislative research organizations) for information on the issue” statement, which the legislators rated more highly. The groups differed on most items pertaining to information-seeking (explore what other states are doing, read scientific reports, read or watch popular media stories, ask research bureaus for information) overall, though they did not differ significantly on the statement “Contact scientific researchers or experts for advice.” We conducted the same analyses by age group solely for outcomes for which the interaction terms between participant group and age were significant (Table 3). Older advocates (≥50 y) more often attended seminars and presentations than older legislators (*P* < .001), but we found no significant difference for younger (<50y) participants (Table 3). For practices related to information use, advocates used research to justify decisions more often than did legislators overall, and older advocates more often talked with colleagues about research on issues important to them and took results of a scientific study into account than older legislators, whereas there was no difference for younger advocates and legislators.

**Table 2:** Comparison of Participating Advocates (N = 77) and Legislators (N = 265) Involved in Cancer Control, Study of Information Use, Information-Seeking, and Research Framing Preferences, United States, 2012–2013

Variable	Advocates	Legislators	Mean Difference Between Scores of Legislators and Advocates	*P* Value^c^
	Mean Score (Standard Deviation)			
**Seeking and Using Information^a^**				
**Using information**				
Use research to justify a decision you made	4.63 (0.10)	4.16 (0.05)	−0.48	<.001
Talk with your colleagues about research on issues important to you	4.37 (0.11)	4.22 (0.06)	−0.15	.20
Take the results of a relevant scientific study into account (when making a decision)	4.29 (0.10)	4.00 (0.05)	−0.28	.01
**Seeking information**				
Explore what other states are doing on the issue	4.12 (0.11)	3.71 (0.06)	−0.41	.001
Read scientific research reports on the issue	3.96 (0.13)	3.38 (0.07)	−0.58	<.001
Read or watch popular media stories on the issue	3.82 (0.13)	2.95 (0.07)	−0.87	<.001
Attend seminars or presentations where research is discussed	3.38 (0.13)	2.91 (0.07)	−0.47	.002
Contact scientific researchers or experts for advice	3.16 (0.14)	2.91 (0.08)	−0.25	.12
Ask research bureaus (external legislative research organization) for information on the issue	2.89 (0.13)	3.34 (0.07)	0.45	.004
**Preferences for Framing of Research Information^b^**				
**Source**				
Research information is unbiased	4.62 (0.10)	4.36 (0.06)	−0.26	.03
Research information is relevant to my constituents/to my organization and those my organization serves	4.62 (0.08)	4.34 (0.05)	−0.28	.004
Research information is delivered to me by someone I know or respect	3.87 (0.10)	4.20 (0.05)	−0.33	.004
Research information supports a position I hold	3.61 (0.13)	3.58 (0.07)	−0.02	.88
**Presentation**				
Research information is understandably written	4.47 (0.08)	4.50 (0.05)	0.04	.71
Research information is presented in a brief, concise way	4.41 (0.09)	4.42 (0.05)	0.01	.90
Research information provides data on the cost-effectiveness of a policy	4.39 (0.09)	4.30 (0.05)	−0.09	.42
Research information tells a story of how a health issue affects my constituents / my organization and those my organization serves	4.32 (0.10)	4.15 (0.05)	−0.17	.12
Research information provides policy options	4.17 (0.10)	4.15 (0.05)	−0.03	.81
**Timeliness**				
Research information is available at the time decisions are being made	4.41 (0.09)	4.43 (0.05)	0.03	.81
Research information deals with an issue that I think is a high priority for state legislative policy action	4.28 (0.09)	4.36 (0.05)	0.09	.40
Research implications are politically feasible at the time I receive them	3.49 (0.13)	3.43 (0.07)	−0.07	.66

a Based on participants’ rating of statements by how often they performed the action (1 = never, 5 = always). Within each subheading, the statements are sorted by the score in the advocates group.

b Based on participants’ ratings of the statements by how much they prioritize the characteristic of the information (1 = low, 5 = high). Within each subheading, the statements are sorted by the score in the advocates group.

c Differences in means were examined by using analysis of variance.

**Table 3:** Comparison of Participating Advocates (N = 76) and Legislators (N = 201) Involved in Cancer Control, by Age, Study of Information Use, Information-Seeking, and Research Framing Preferences, United States, 2012–2013^a^

Variable	Aged <50 Years			Aged ≥50 Years		
	Advocates (n = 41), Mean Score (SD)	Legislators (n = 40), Mean Score (SD)	*P* Value^b^	Advocates (n = 35), Mean Score (SD)	Legislators (n = 161), Mean Score (SD)	*P* Value^b^
**Seeking and Using Information^c^**						
**Using information**						
Talk with your colleagues about research on issues important to you	4.20 (0.14)	4.40 (0.14)	.30	4.56 (0.16)	4.19 (0.07)	.04
Take the results of a relevant scientific study into account (when making a decision)	4.05 (0.16)	4.10 (0.16)	.82	4.54 (0.13)	3.98 (0.06)	<.001
**Seeking information**						
Attend seminars or presentations where research is discussed	2.98 (0.19)	2.93 (0.19)	.85	3.80 (0.18)	2.91 (0.09)	<.001
**Preferences for Framing of Research Information^d^**						
**Source**						
Research information is delivered to me by someone I know or respect	3.58 (0.14)	4.40 (0.14)	<.001	4.17 (0.14)	4.19 (0.07)	.88
**Timeliness**						
Research information deals with an issue that I think is a high priority for state legislative policy action	4.02 (0.11)	4.48 (0.11)	.006	4.56 (0.13)	4.37 (0.06)	.18

a This table includes outcomes for which the interaction terms between participant group and age were significant. Within each subheading, the statements are sorted by score in the advocates group.

b Differences in means were examined by using analysis of variance.

c Participants were asked to rate the statements based on how often they perform the action described in the statement (1 = never, 5 = always).

d Participants were asked to rate the statements based on how much they prioritize the characteristic of the information (1 = low, 5 = high).

For the items related to preferences for research information framing, there were significant differences (3 out of 4 items) between the 2 groups’ preferences for the source of research information. Advocates put a higher priority than did legislators on research information that was unbiased and relevant to their organization and constituents overall. Younger legislators put a higher priority on information that was delivered to them by someone they know or respect (4.40 ranking on the 5-point scale) than did younger advocates (3.58), whereas both older advocates (4.17) and older legislators (4.19) found this to be important. Advocates and legislators put a similar priority on research information that supports the position they hold. Legislators’ and advocates’ preferences were similar for the presentation (research information being understandably written, presented briefly and concisely, containing cost-effectiveness data, telling a story of relevance to constituents, and providing policy options) and most timeliness issues (research information is available at the time of decision-making and containing implications that are politically feasible). Whereas younger legislators prioritized research information dealing with high-priority issues higher than younger advocates, we found no difference between older legislators and older advocates.

## Discussion

We examined differences in how state-level legislators and advocates working in cancer control use research information, specifically comparing information-seeking, information use, and preferences for framing of research information, and found that advocates overall used and sought information more often than legislators did, but legislators more often used legislative research organizations for information. Though both groups gave high rankings to information that is well presented and timely, we found differences in how the 2 groups prioritized the source of information. Legislators emphasized relationships, whereas advocates rated objectivity and relevance to constituents more highly. Several of the differences between advocates and legislators were associated with age.

Studies such as ours are important for developing and tailoring knowledge translation strategies for policy makers and advocates. Such studies also begin to fill a gap in research regarding knowledge translation among advocacy organizations. Our findings show that both groups valued research evidence and used research reports and studies in policy making, though advocates more often than legislators, but both groups infrequently contacted researchers or experts for advice. Both groups preferred research information that is understandable, concise, relevant, actionable, timely, and includes cost-effectiveness data. Younger advocates, who make up most of the advocates group, did not prioritize framing of information by someone they know or trust, so integration of this item into translation strategies tailored to advocates may be of less importance. However, for legislators, who prioritize this way of receiving research information, knowledge translation strategies should include the establishment of a relationship of trust with the legislator by the researcher or by another actor who is able to serve as an intermediary. Findings of previous studies indicate that these relationships represent a long-term investment, requiring considerable time, effort, and in-person communication (13,19,20). In addition, because legislators reported often using internal legislative research bureaus to obtain information, researchers and advocates should develop strategies for more effective evidence sharing with these entities. Advocates placed a higher priority on information that was unbiased and relevant to their constituents than did legislators; therefore, ensuring that the research evidence is generalizable to these constituencies and including local data and success stories in dissemination materials are important.

This study’s findings are largely in line with existing literature on knowledge translation among legislators and other policy makers. A qualitative study of Wisconsin and New York state legislators found that they value research evidence and find evidence useful when it is credible, accessible to them, and available when decisions are made (16). The study found that legislators would like to receive information in a format that provides opportunity for interaction, including seminars, interactions with experts, and discussion with colleagues (16). A large amount of research exists on barriers and facilitators to the use of evidence, though little has been conducted with advocates (19). The top 5 barriers and facilitators to the use of evidence by policy makers are availability and access to research; clarity, relevance, and reliability of research findings; collaboration and relationships between policy makers and researchers; and policy maker research skills (19). A qualitative study with nutrition and obesity researchers active in policy dissemination found that cultivating relationships with policymakers, use of intermediaries (eg, professional associations or nonprofit organizations that aim to improve knowledge sharing between producers and users of knowledge) in this work, and providing relevant policy communication training to researchers are promising strategies for success in knowledge translation into policy (20). Moreland-Russell et al qualitatively examined how legislative testimony influences the actions of legislators in our study and found that it influences awareness of issues and legislative decision-making and that presenter credibility, knowledge, and expertise increase the influence of testimonies (17).

Eyler et al qualitatively examined how the advocates in our sample communicate with policy makers, what barriers they experience, and what strategies they find useful (13) and found that advocates’ perceptions of what works in communicating research to policy makers matches the preferences reported by state legislators in our study. Advocates found that developing and maintaining professional relationships with policy makers is essential and takes long-term commitment, and that policy makers like to receive credible, relevant, and timely information that is understandable and concise and includes cost information (13). Other research, dealing with obesity prevention policy, showed that advocates are more aware of and able to communicate policy-relevant evidence (21) though few studies outside of our project (15) have examined how advocates prefer to receive research information.

Knowledge translation strategies tailored to advocacy organizations are particularly important, because advocates often act as information channels to policy makers and can support evidence-based policy making through activities not usually carried out by researchers (12,13,22,23). Advocates routinely build coalitions around common policy issues and coordinate collective messaging and exchange of information among actors (12) to ensure that policy makers hear the same coherent meta-message around a problem. They are also skilled at selecting the appropriate messengers to convey information to policy makers (13). Though researchers can and do participate in the policy making process, they encounter several barriers to doing so (23,24), and partnerships with advocacy organizations may address some of these barriers. 

The findings from our study may not be equally generalizable to all states or policy areas. Policy networks (ie, patterns of interaction between public and private actors in policy making) may differ between states, and these networks inform the degree to which policy activities are coordinated, to what extent new actors or new ideas are allowed to enter into the policy decision-making sphere, and what kind of information-framing is necessary (25,26). For example, in policy networks where legislators or their staff are engaged with other actors through regular contact (27), dissemination strategies may include working through these relationships instead of establishing new ones. In networks that insulate policy decision making from issue interest groups and are thus more resistant to policy change (25), researchers must take these network structures into account and tailor their dissemination strategies accordingly.

Differences exist between policy topic areas in how acceptable they are perceived to be by policy makers (28) or how likely they are to be enacted (29), and different policy issues are associated with different policymaker research-framing preferences (18). In addition, high profile policy issues accompanied by high interest from media and the public may lead to more contested relationships between policy actors (25), changing the context in which research translation takes place. Finally, enactment of evidence-based policy is more likely in the presence of an existing policy on the topic (29), which varies by policy and state. These differences contribute to the complexity of translating research into policy and should be taken into account when developing translation strategies.

This study addresses a gap in the literature on translation of cancer control research into policy and used robust measures of information seeking and information use and preferences for research-framing. However, some limitations must be noted. Different sampling procedures were used to select legislators and advocates, which may have reduced comparability of the 2 groups. We used a comprehensive sampling frame to recruit legislators, whereas we did not sample advocates based on a comprehensive list. In addition, we sampled advocates working on cancer and cancer risk factor issues from the outset, whereas we selected legislators who were prioritizing these issues in their work on the basis of their questionnaire responses. In addition, our study’s response rates (54% for legislators, 36% for advocates) may reduce the generalizability of our findings, although our response rate for legislators was higher than that achieved in other policy-related studies (10,30). It is also possible that in both groups a social desirability bias led to higher ratings on questionnaire items related to research evidence, because the survey was part of an academic research project. Finally, our study would have been strengthened by collection of additional qualitative data to examine how and where participants accessed research, or to assess information-seeking and use beyond participant self-report to validate our measures.

Our study provides insights for development of knowledge translation strategies to enhance evidence-based policymaking for cancer control that are tailored to state-level legislators and advocates. When working with both groups, research information should be understandable, concise, relevant, actionable, and timely, and should include cost-effectiveness data. Translation strategies for legislators should include partnering with individuals and groups that have existing personal relationships with legislators and their staff and more effective evidence-sharing with legislative research bureaus. For advocates, ensuring that the research evidence is generalizable to their constituencies and including local data and success stories in dissemination materials are important. In addition, though a growing knowledge base exists on how to disseminate research to policy makers, few studies identify strategies for knowledge translation to advocates. Future research efforts should examine how these key actors in the policymaking process can be more effectively engaged to promote evidence-based policy making.
